# Therapeutic effects of matrine derivate MASM in mice with collagen-induced arthritis and on fibroblast-like synoviocytes

**DOI:** 10.1038/s41598-017-02423-7

**Published:** 2017-05-26

**Authors:** Yuming Zou, Quan Li, Denghui Liu, Jia Li, Qing Cai, Chao Li, Qingjie Zhao, Weidong Xu

**Affiliations:** 1 Department of Orthopedics, Changhai hospital, the first affiliated hospital of the Second Military Medical University, Shanghai, 200433 P.R. China; 2 Orthopedics Department, Renji Hospital, School of Medicine, Shanghai Jiaotong University, Shanghai, 200127 P.R. China; 3 Department of Rheumatology, Changhai hospital, Second Military Medical University, Shanghai, 200433 P.R. China; 4 Department of Organic Chemistry, School of Pharmacy, the first affiliated hospital of the Second Military Medical University, Shanghai, 200433 P.R. China

## Abstract

MASM is a matrine derivate that exhibits a number of pharmacological effects, including immunosuppressive activity and anti-inflammatory properties. In this study, the mechanisms underlying the therapeutic efficacy of MASM in the treatment of rheumatoid arthritis were investigated using DBA/1 mice with collagen-induced arthritis (CIA) and fibroblast-like synoviocytes derived from rheumatoid arthritis patients (RA-FLS). We demonstrated that MASM markedly attenuated the severity of arthritis in CIA mice. The therapeutic effects were associated with ameliorated joint swelling and reduced bone erosion and destruction. Furthermore, the administration of MASM suppressed the expression of pro-inflammatory cytokines (TNF-α, IL-1β, IL-6). *In vitro*, MASM inhibited the expression of pro-inflammatory cytokines (TNF-α, IL-6, IL-8) and matrix metalloproteinases (MMP-1, MMP-3 and MMP-13) by inhibiting both the phosphorylation of MAPKs and the activation of NF-κB in IL-1β-stimulated RA-FLS. Additionally, MASM could induce apoptosis of RA-FLS via mitochondrial and Akt signaling pathways in human RA-FLS. These findings suggest that MASM could attenuate arthritis severity in CIA mice at least partially by blocking the phosphorylation of MAPKs and the activation of NF-κB and by inducing apoptosis in RA-FLS. MASM could be a potent therapeutic agent for the treatment of RA.

## Introduction

Rheumatoid arthritis (RA) is a chronic inflammatory autoimmune disease and is characterized by leukocyte infiltration and synovial hyperplasia that causes chronic joint inflammation as well as the subsequent erosion of cartilage and bone^1, 2^. Fibroblast-like synoviocytes (RA-FLS) play a key role in RA by producing inflammatory mediators that initiate and perpetuate inflammation as well as proteases that contribute to cartilage and bone destruction. Additionally, the interaction between RA-FLS and cells of the immune system or resident joint cells could lead to the promotion of ongoing inflammation and tissue destruction^3, 4^. Additionally, RA-FLS are relatively resistant to apoptosis and develop a unique aggressive phenotype by forming hyperplastic synovial pannus tissue^3, 5^.

Matrine is an active alkaloid compound that is isolated from the traditional Chinese herb *Sophora flavescens* and has a wide spectrum of biological properties, such as anti-inflammatory^6–8^, antiviral^9^, anti-cancer^10–12^, anti-fibrotic^13^ and anti-dementia^14^ properties. However, until now matrine has not been developed into a drug because of its low therapeutic efficacy. A series of matrine derivatives have been designed and synthesized, including MASM [(6aS, 10 S, 11aR, 11bR, 11cS)210-Methylamino-dodecahydro-3a, 7a-diaza-benzo (de)anthracene-8-thione], that have exhibited better pharmacological functions than matrine, as we reported in our previous studies^15–17^. MASM was found to possess potent anti-inflammatory^16, 18^, anti-fibrotic^17^ and radio protective activity^19^.

In the present study, we found that MASM significantly alleviated arthritis in collagen-induced arthritis (CIA) mice. Further investigation revealed that MASM could significantly suppress inflammatory responses by inhibiting both the phosphorylation of MAPKs and the activation of NF-κB. Additionally, MASM induces apoptosis in human RA-FLS by activating the mitochondrial apoptosis pathway and inhibiting the Akt signaling pathway.

## Results

### Suppression of synovial inflammation and joint destruction by MASM in mice with CIA

**Figure 1:**
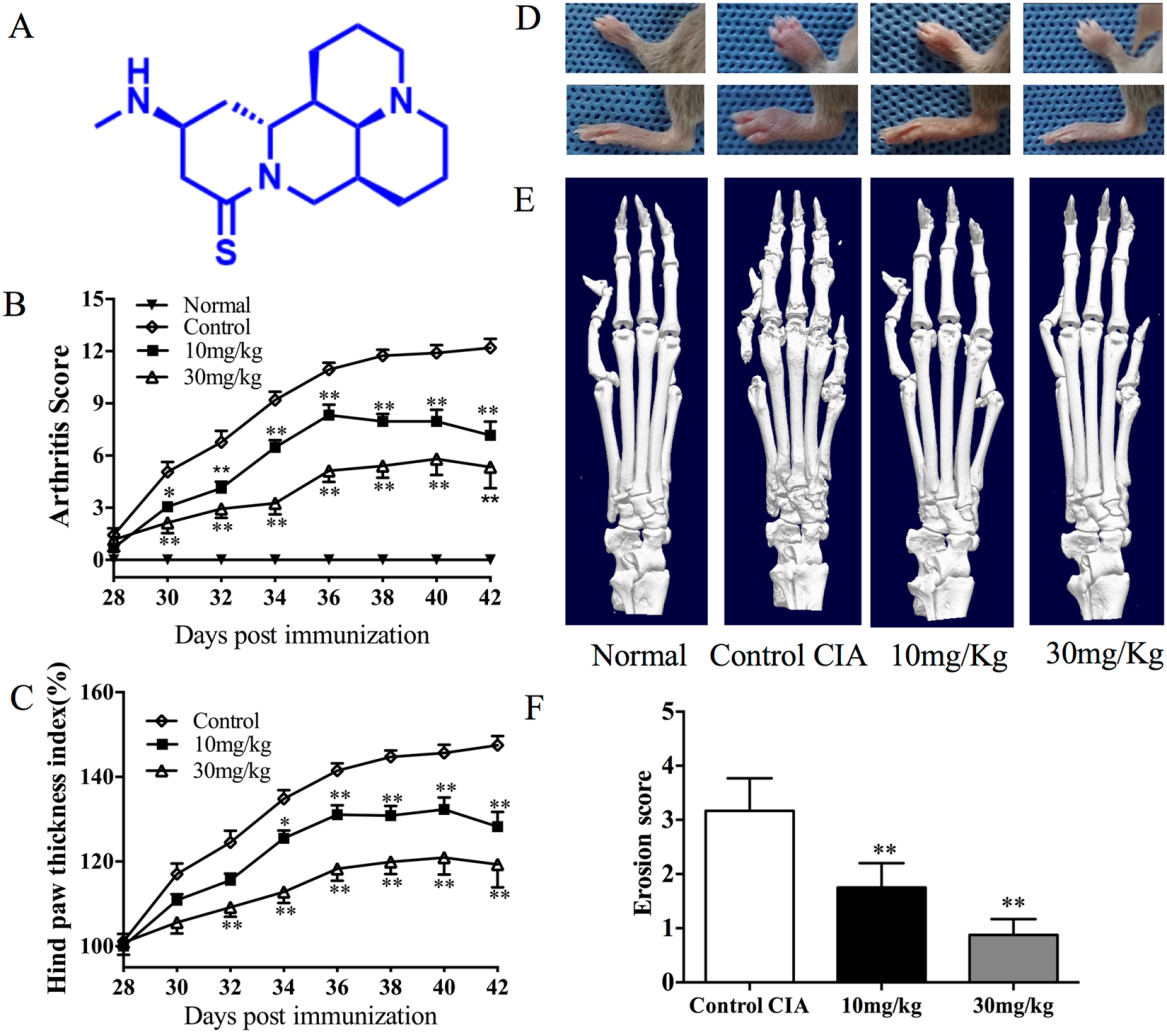
Effects of MASM administration on mice with CIA. Mice were injected with saline or the indicated concentrations of MASM on day 22 (1 day after booster immunization on day 21). (**A**) The chemical structure of MASM. The mean clinical arthritis scores (**B**) and the hind paw thicknesses (**C**) were determined on the indicated days after the primary immunization. (**D**) Representative photographs of the hind paws of CIA mice on day 42. (**E**) Representative photographs of 3D reconstruction of micro-computed tomography of the hind paws (right) of mice with CIA. (**F**) Bone Erosion scores in CIA groups. Values in (**B**), (**C**), (**F**) are mean ± SEM from a representative experiment (n = 10~15). ^*^P < 0.05, ^**^P < 0.01 versus CIA mice treated with saline.

The chemical structure of MASM is shown in Fig. 1A. We assessed the therapeutic effects of MASM in mice with CIA. The clinical scores were significantly lower in the MASM treatment group than in the control group and exhibited a dose-dependent behavior (Fig. 1B). Furthermore, hind paw thickness was measured to analyze the beneficial effects of MASM, and the results were correlated with clinical scores (Fig. 1C). MASM attenuated the swelling, erythema, and joint rigidity of the paws in mice with CIA (Fig. 1D). A 3D reconstruction of a micro-CT scan of hind paws showed typical changes in mice with CIA, which are characterized by articular destruction, joint displacement, and irregular bony proliferation. However, MASM treatment alleviated bone destruction. The mean erosion scores also demonstrated markedly less bone destruction in the MASM treatment groups (Fig. 1E and F). Larger number of regional enlarged representative 3D reconstructions of the hind paws are provided in Figure S1.

### Suppression of histopathological abnormalities by MASM

**Figure 2:**
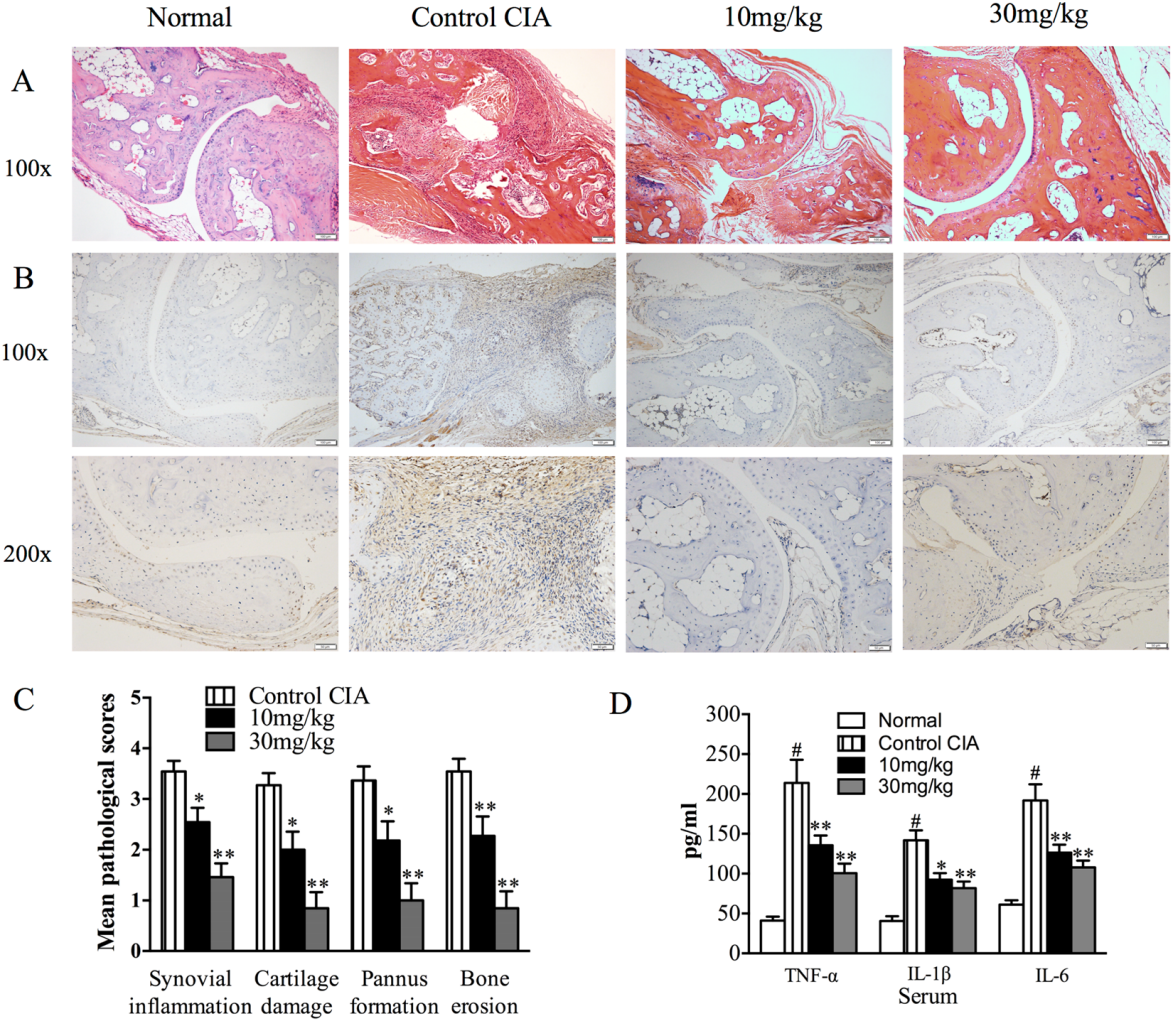
Effects of MASM administration on histopathological abnormalities and the production of pro-inflammatory cytokines in mice with CIA. (**A**) Hematoxylin and eosin (H&E) staining of the hind paws from the indicated different four groups. Original magnification x100. (**B**) Local tumor necrosis α (TNF-α) expression in the hind paws tissues was measured by immunohistological staining. Original magnification x100 and x200. (**C**) Pathological scores determined as described in Materials and Methods. (**D**) The serum levels of TNF-α, IL-1β and IL-6 were assessed by specific enzyme-linked immunosorbent assay (Elisa). Bars show the mean ± SEM (n = 10–15 mice per group). ^#^P < 0.01 compared with normal group. ^*^P < 0.05, ^**^P < 0.01 versus the control group.

Severe synovial inflammation, cartilage damage, pannus formation, and bone erosion were observed in the control CIA mice. By contrast, ankle joints from MASM-treated mice with CIA showed remarkable improvements in histopathological findings (Fig. 2A). Consistently, the histological scores revealed that synovial inflammation, cartilage damage, pannus formation and bone erosion were significantly attenuated by MASM in CIA mice (Fig. 2C). Larger number of representative histological pictures of hind paws are provided in Figure S2.

### Suppression of the production of pro-inflammatory cytokines

It is well known that pro-inflammatory cytokines, such as TNF-α and IL-1β, play key roles in the pathogenesis of RA^20^. The cytokine levels were therefore measured by immunohistochemical staining using joint tissues and ELISA using serum obtained on day 42. Immunohistochemical staining indicated that MASM significantly suppressed TNF-α production in joint tissues (Fig. 2B). Consistent with the immunohistochemical staining findings, the serum level of TNF-α was significantly lower in the MASM-treated group compared to the control CIA group. The same results were found in the serum levels of IL-1β and IL-6 (Fig. 2E).

### MASM suppressed the IL-1β-induced production of pro-inflammatory cytokines and proteases in RA-FLS and induced apoptosis of RA-FLS

**Figure 3:**
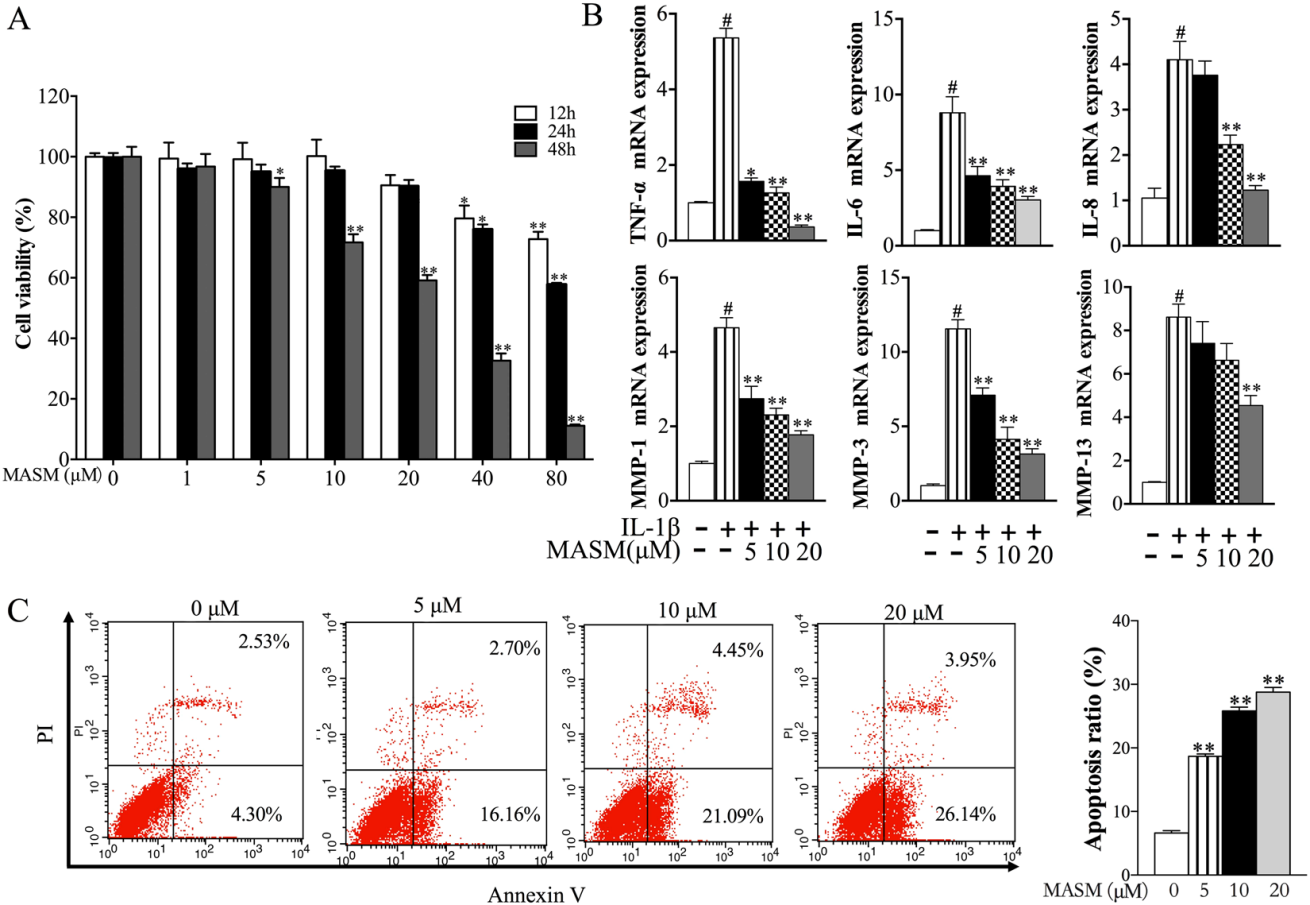
Effects of MASM on the IL-1β-induced expression of pro-inflammatory cytokines and proteases in human RA-FLS and its effect on apoptosis of human RA-FLS. (**A**) Effects of MASM on cell viability. Cells were seeded in a 96-well plate (6 × 10^3^/well) and cultured with various concentrations of MASM (0, 5, 10,20, 40, 80, 160 μM) for 12 h, 24 h and 48 h. (**B**) Effects of MASM on expression of pro-inflammatory cytokines (TNF-α, IL-1β, and IL-6) and proteases (MMP-1, MMP-3, MMP13) in human RA-FLS. Human RA-FLS were pre-treated with different concentrations of MASM (0–20 μM) for 1 hour and then treated with 2 ng/ml IL-1β for another 24 h. (**C**) Effects of MASM on apoptosis of human RA-FLS. Human RA-FLS were pre-treated with different concentrations of MASM (0–20 μM) for 1 hour and then treated with 2 ng/ml IL-1β for another 48 h. Apoptosis in human RA-FLS was assessed using Annexin V-FITC/PI double staining and analyzed by flow cytometry. The bars show the mean ± SEM from 3 independent experiments. (**A**) ^*^P < 0.05, ^**^P < 0.01 versus RA-FLS in the absence of MASM treatment. (**B**,**C**) ^#^P < 0.01 compared to the control RA-FLS. ^*^P < 0.05, ^**^P < 0.01 versus RA-FLS stimulated with IL-1β in the absence of MASM.

RA-FLS are key effector cells in the pathogenesis of RA, and they play a central role by producing cytokines that perpetuate inflammation and proteases that contribute to cartilage destruction. Additionally, RA-FLS are resistant to apoptosis and develop a unique aggressive phenotype that increases invasiveness into the extracellular matrix and further exacerbates joint damage^3^. Thus, we investigated the effects of MASM on the cytokine and protease production in IL-1β induced RA-FLS. Additionally, we examined whether MASM could regulate the apoptosis of RA-FLS. Primarily, we tested the effects of MASM on the cell viability of RA-FLS. The RA-FLS were exposed to MASM at different concentrations and for different periods of time, and the cell viability was measured using a CCK8 assay. As shown in Fig. 3A, MASM (20 μM) had no significant effect on the cell viability of RA-FLS at 12 and 24 h but not 48 h. To investigate the possible inhibitory effects of MASM on IL-1β-induced RA-FLS function, RA-FLS were pretreated with or without increasing the concentration of MASM ranging from 5 to 20 μM for 1 h and then exposed to IL-1β (2 ng/ml) for 24 h. Our results indicated that MASM could significantly inhibit the production of pro-inflammatory cytokines (TNF-α, IL-6, and IL-8) and MMPs (MMP-1, MMP-3, and MMP-13) in RA-FLS in a dose-dependent manner (Fig. 3B). To determine the effects of MASM on apoptosis of RA-FLS, the apoptosis ratio of MASM treated RA-FLS was evaluated by Annexin V/PI double staining. The finding shows that MASM (5 to 20 μM, 48 h) could significantly induce apoptosis of RA-FLS in a dose-dependent manner compared to the control group treated with IL-1β alone (Fig. 3C).

### Effects of MASM on IL-1β-induced MAPK and NF-κB signaling pathways

Two principal pathways that are activated in the pathogenesis of RA are the mitogen-activated protein kinases (MAPKs) and nuclear factor-kappa B (NF-κB) pathways^21–24^. Enhanced MAPK signaling has a demonstrated role in the increased production of cytokines and MMPs relative to the pathogenesis of RA. As an important transcription factor, NF-κB activation plays a key role in regulating synovial inflammation, hyperplasia, and joint destruction.

**Figure 4:**
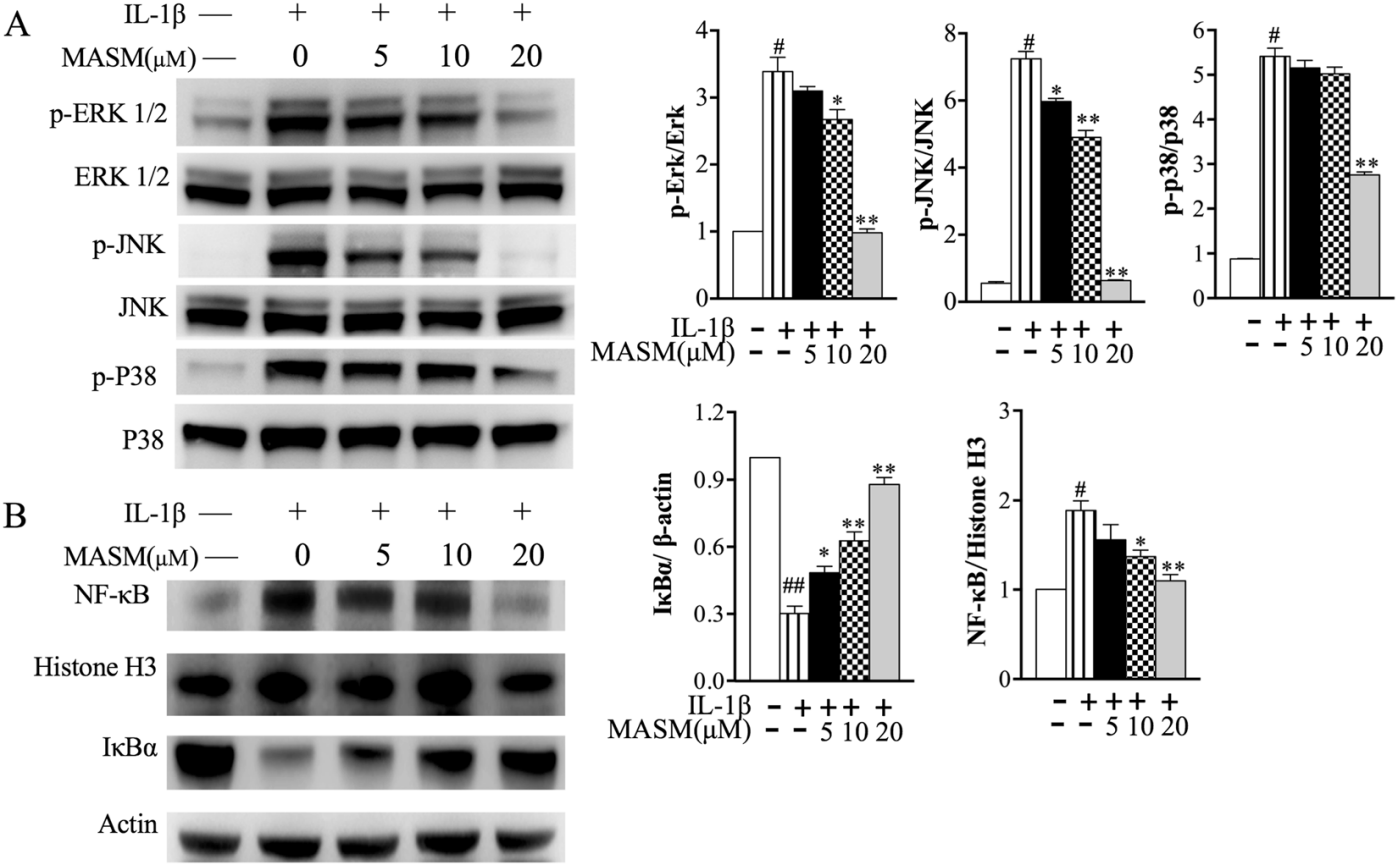
Effects of MASM on IL-1β-induced phosphorylation of MAPKs and the activation of NF-κB in human RA-FLS. Human RA-FLS were pre-treated with different concentrations of MASM (0–20 μM) for 1 hour and then treated with 2 ng/ml IL-1β for another 30 min. (**A**) Western blotting was performed to detect the total and phosphorylated levels of JNK, ERK1/2 and p38 in the RA-FLS with the indicated treatments. (**B**) Translocation of NF-κB/p65 to the nucleus and IκBα degradation in the cytoplasm were also determined by western blotting. The band intensity of the western blot bands was normalized to loading controls, and the values are the mean ± SEM of three independent experiments. β-actin and Histone H3 were used as loading controls for cytoplasmic and nuclear proteins, respectively. ^#^P < 0.01 compared to control RA-FLS. ^*^P < 0.05, ^**^P < 0.01 versus RA-FLS stimulated with IL-1β in the absence of MASM.

To further characterize the mechanism through which MASM exerts inhibitory effects on RA-FLS, we examined whether MASM regulates these transduction pathways. Human RA-FLS were pre-treated with different concentrations of MASM (5–20 μM) for 1 hour before stimulation with IL-1β (2 ng/ml) for another 30 min. Then, the levels of the phosphorylation of mitogen-activated protein kinases, including ERK, JNK and p38, and the level of NF-κB in the nucleus as well as the cytoplasmic IκBα were analyzed using western blot. IL-1β treatment for 30 min significantly induced the phosphorylation of ERK1/2, p38 and JNK. The phosphorylation of Erk1/2, p38, and JNK was significantly inhibited by MASM in a concentration-dependent manner (Fig. 4A). The nuclear translocation of NF-κB/p65 and the degradation of cytoplasmic IκBα were also observed in RA-FLS stimulated by IL-1β and were dose-dependently decreased by MASM (Fig. 4B).

### Effects of MASM on mitochondrial apoptosis pathway-related proteins

**Figure 5:**
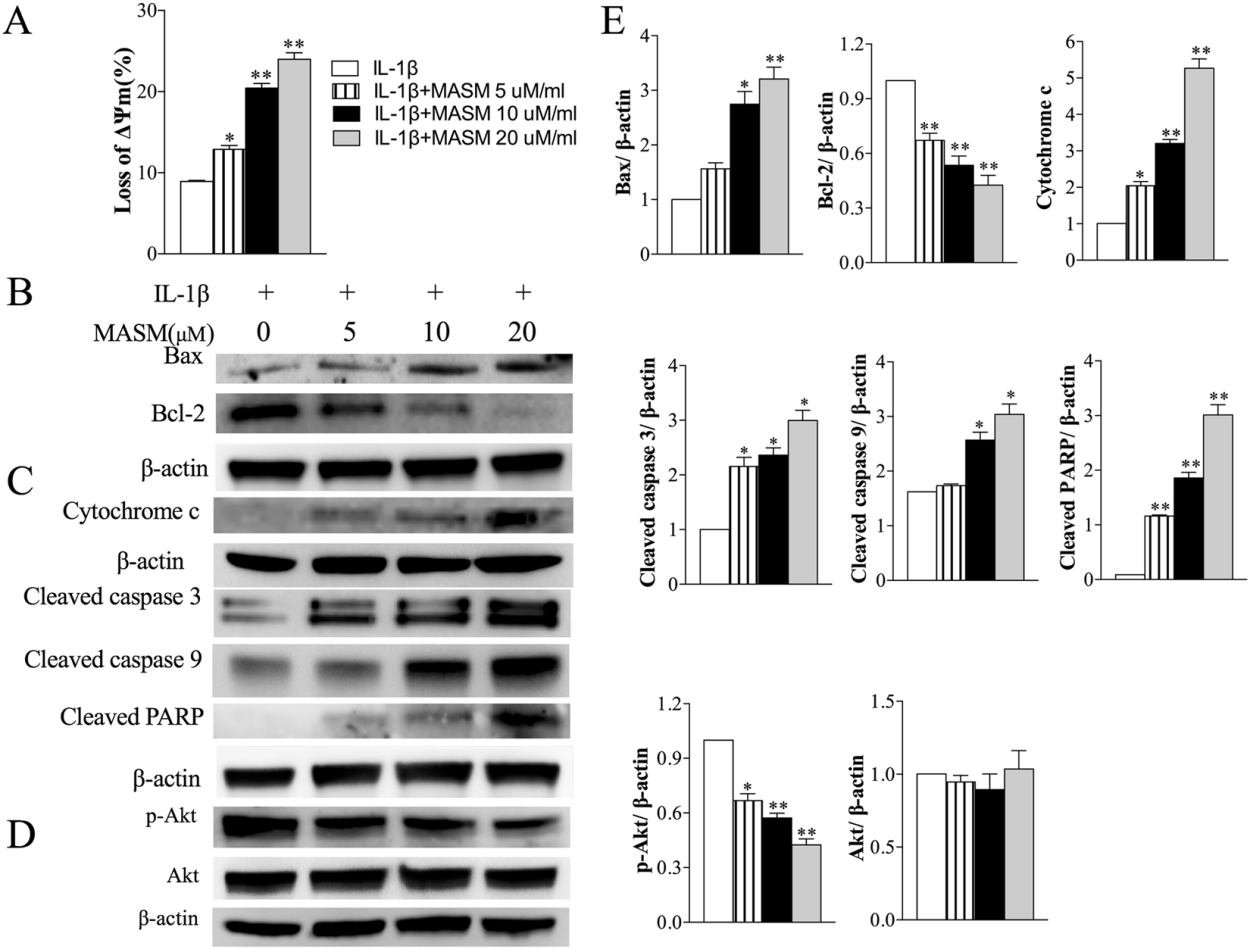
The effects of MASM on the expression of apoptosis-related factors in human RA-FLS. Cells were pretreated with different concentrations of MASM (0–20 μM) for 1 h, and then 2 ng/ml IL-1β was added for another co-stimulation of 48 hours. (**A**) After the indicated treatment, the mitochondrial membrane potential was measured by the JC-1 dye, the mean JC-1 fluorescence intensity was detected by FASC analysis. The quantification of the result is shown. (**B**) Western blot analysis of Bax, Bcl-2, Cytochrome C, Cleaved caspase 3, cleaved caspase 9 and Cleaved caspase PARP expression. (**C**) Western blot analysis p-Akt, Akt, (**D**) Band intensities was normalized to β-actin. Data are shown as the mean ± SEM (n = 3) from three independent experiments. ^*^P < 0.05, ^**^P < 0.01, relative to control RA-FLS.

As is shown in Fig. 3C, MASM induced apoptosis in RA-FLS in a dose-dependent manner. To further determine the apoptotic pathway that was activated in response to the treatment of MASM, we detected the mitochondrial membrane potential in RA-FLS exposed to different concentrations of MASM. Changes in the mitochondrial membrane potential were identified by the JC-1 dye. The results indicated that MASM induced mitochondrial depolarization in RA-FLS in a concentration-dependent manner (Fig. 5A). Consistently, western blot analysis demonstrated that the levels of pro-apoptotic protein Bax were markedly up-regulated but that anti-apoptotic Bcl-2 was significantly reduced by MASM in RA-FLS in a dose-dependent manner (Fig. 5B). Bcl-2 then increased the release of cytochrome C into the cytoplasm and induced the cleavage of caspase-9, caspase-3, and PARP (Fig. 5C). The band intensities were normalized to β-actin (Fig. 5E).

### Effects of MASM on the activation of Akt signaling pathways

The phosphorylation of Akt protects RA-FLS from apoptosis^25, 26^. The effects of MASM on the activation of Akt were analyzed by western blot. Our results indicated that the phosphorylation of Akt was inhibited by MASM but that the expression of total Akt was not affected (Fig. 5D). The band intensity was normalized to β-actin (Fig. 5E).

## Discussion

In this study, we demonstrated that MASM effectively suppressed inflammatory responses and joint destruction in CIA mice. Furthermore, an *in vitro* study indicated that MASM inhibited the activation of MAPKs and NF-κB in RA-FLS and induced apoptosis of RA-FLS via the mitochondrial apoptosis and Akt signaling pathways.

Accompanied by synovial hyperplasia, joint inflammation and the subsequent destruction of cartilage and bone, RA is the most common inflammatory arthritis. It affects approximately 1.0% of adults and is a major source of disability^2, 23, 24^. Despite advances made over the past two decades in the treatment of RA, almost 20–50% of all such patients show an inadequate response or have to discontinue therapy because of intolerance or adverse events; as a result, there is an urgent need for new therapeutic agents^27, 28^.

Matrine is an alkaloid isolated from the Chinese herb *Sophora flavescens*, which possesses a wide scope of biological activities. It was reported to inhibit the production of inflammatory mediators, such as TNF-α and IL-6 in murine macrophages^8^ and in mice with acute lung injury^29^. Recently, matrine was reported to suppress inflammatory responses and alleviate arthritis in CIA rats^7^. MASM, which is a synthesized derivative of matrine, exhibited better pharmacological activities but had fewer side effects than matrine^15–19, 30^. All of these effects raised the possibility that MASM could be a potential agent for treating rheumatoid arthritis.

In the present study, we demonstrated that MASM effectively ameliorated arthritis in CIA mice. Histological analysis and a micro-CT scan of the hind paws further verified that synovial inflammation, cartilage damage, pannus formation, and bone erosion were significantly attenuated by MASM treatment. In CIA mice, various cytokines had crucial roles in activating immune and inflammatory cells, especially the pro-inflammatory cytokines, which favored the induction of autoimmunity, chronic inflammation and subsequent joint destruction^31^. Our results showed that 20 days of treatment with MASM significantly suppressed the systematic levels of pro-inflammatory cytokines, such as TNF-α, IL-1β and IL-6, and the local expression of TNF-α *in vivo*.

RA-FLS are a key component of invasive rheumatoid synovium and have a major role in the initiation and perpetuation of destructive joint inflammation^3, 5, 32^. IL-1β is the predominant pro-inflammatory cytokine associated with RA, and it can stimulate the proliferation of FLS and increase the production of inflammatory mediators^33^. In light of its effects, IL-1β has been widely used to mimic arthritis in *in vitro* studies^34^. To further explore the possible pharmacological mechanism of MASM in the treatment of RA, we investigated the effects of MASM on IL-1β-stimulated human FA-FLS *in vitro*. The findings showed that MASM significantly inhibited the expression of pro-inflammatory cytokines, such as TNF-α, IL-6 and IL-8, and the expression of MMPs, such as MMP-1, MMP-3, and MMP-13, in IL-1β-stimulated human FA-FLS.

It has been well documented that the two principal pathways activated in the pathogenesis of RA are the MAPKs and NF-κB signaling pathways, which are key regulators of cytokine and metalloproteinase production^21–24^. Targeting MAPKs or NF-κB signaling pathways is an effective therapeutic strategy^24, 35–39^. In the present study, IL-1β stimulation markedly induced both the phosphorylation of MAPKs and the translocation of NF-κB into the cell nucleus in RA-FLS, whereas MASM treatment considerably inhibited the phosphorylation of MAPKs, including JNK, ERK and p38, and the translocation of NF-κB into the nucleus, especially at a high concentration of 20 μM. The current findings agreed with previous studies that showed MASM inhibited the MAPK and NF-κB signaling pathways. Hu *et al*.^16^ reported that MASM significantly suppressed the TNF-α production and NF-κB transcriptional activity in LPS-stimulated RAW264.7 cells. Xu *et al*.^18^ showed MASM could inhibit LPS-induced activation of NF-κB and MAPK pathways in RAW264.7 cells and could prolong survival, attenuate inflammation, and reduce organ injury in murine established lethal sepsis. Li *et al*.^19^ reported that MASM provides a protective effect in total-body irradiated (TBI) rats, which partially arises via the modulation of MAPK pathways. Qian, *et al*.^30^ reported the inactivation of Erk signaling in hepatocellular carcinoma cells. Some of the discrepancies that exist can be attributed to differences in MASM exposure time or a different cell type.

RA-FLS are relatively resistant to apoptosis both *in vivo* ^40^ and *in vitro* ^41, 42^. The imbalance between the proliferation and apoptosis of RA-FLS could lead to the formation of a hyperplastic pannus. Therapies aimed at inducing apoptosis of RA-FLS could be valuable for RA treatment^5, 43^. The anti-cancer and apoptosis-inducing properties of matrine are well documented^10, 12, 44, 45^. A previous study investigated the effects of MASM on rat hepatocytes and hepatic stellate cells (HSCs). MASM treatment dose-dependently (5~20 μM for 72 hours) inhibited the proliferation of HSCs and activated HSCs but did not activate primary rat hepatocytes; the retardation in proliferation could not be attributed to an increase in cell apoptosis^17^. In the present study, we investigated whether MASM had an effect on the apoptosis of RA-FLS, and the expression of apoptosis-related factors was examined. The results showed that MASM induced the apoptosis of RA-FLS in a dose-dependent manner. The mitochondrial apoptotic pathway and PI3Kinase/Akt signaling pathway, which were both involved in the resistance of RA-FLS to apoptosis^25^ were also examined. MASM induced mitochondrial depolarization in RA-FLS, which inhibited the expression of the anti-apoptotic protein Bcl-2 and enhanced the expression of pro-apoptotic protein Bax. Consistently, MASM increased the release of cytochrome c and enhanced the cleavage of caspase-3, caspase 9 and PARP. In addition, p-Akt is a well-known anti-apoptosis protein and a major downstream kinase of PI3K, and it plays an important role in the reduced apoptosis of RA-FLS^25, 26^. Previously, MASM was shown to exhibit potent anti-inflammatory, immunomodulatory and inhibitory activity against liver fibrosis *in vitro* and *in vivo*, along with the reduction of Akt phosphorylation^15–17^. Accordingly, we examined the p-Akt level in RA-FLS after treatment with MASM, and the results showed that the expression of p-Akt was reduced in a dose-dependent manner. Collectively, these results revealed that MASM-mediated apoptosis of RA-FLS could be conducted via the mitochondrial and Akt signaling pathways.

This study had several limitations. First, the timing of MASM administration had an impact on its efficacy. In the current study, MASM was administered early after the disease onset. Whether MASM can control disease progress during the active phase of arthritis has yet to be investigated. Second, the optimal duration of administration and the durability of the MASM effects require clarification, and the occurrence of any adverse effects during long-term treatment must be documented. Third, apart from possible mechanisms, we argued that MASM could be used in the treatment of RA, but we did not exclude the possibility of unidentified effects of MASM on other mechanisms. For example, the dendritic cell (DC) maturation process is a crucial step for the development of T cell immune responses and immune tolerance, which likely has a role in the pathogenesis of RA, could be affected. MASM could suppress LPS-induced phenotypic and functional maturation of murine bone marrow-derived dendritic cells^15^. This suppression could be another possible mechanism that MASM performs as part of its treatment effects in RA, but it needs further investigation.

In summary, we determined the effects of a novel matrine derivate (MASM) that significantly reduced the severity of inflammation and joint destruction in CIA mice. *In vitro* analyses demonstrated that MASM could suppress the expression of inflammatory mediators by inhibiting MAPK and NF-κB pathways and inducing apoptosis of RA-FLS via the mitochondrial and Akt signaling pathways in human RA-FLS. Because of its advantages over matrine, MASM has the potential to be used as a novel therapeutic agent for treating human RA.

## Materials and Methods

### Animals

Specific pathogen-free, 7~8-week-old male DBA/1 mice were purchased from the Vital company (Beijing, China). The mice were maintained in a laminar flow cabinet at the Center Laboratory of Changhai Hospital under standard laboratory conditions with respect to temperature, pressure, and humidity. Food and water were sterilized by 60 Co γ-irradiation and high pressure, respectively. All animal procedures were carried out according to the recommendations in the Guide for the Care and Use of Laboratory Animals of the National Institutes of Health. All experimental animals used in this study were maintained under the protocol approved by the Ethics of Animal Experiments of Second Military Medical University (Shanghai, China).

### Reagents

The matrine derivative MASM [(6aS, 10 S, 11aR, 11bR, 11cS)210-Methylamino-dodecahydro-3a, 7a-diaza-benzo (de)anthracene-8-thione] (purity > 99%) was synthesized via a classical Michael addition and characterized as reported previously^16^.

### Induction of CIA

To test the therapeutic effects of MASM, a CIA model was conducted according to a previous protocol^46^. The mice were immunized intradermally by 100 mg of chicken type II collagen (CII; Chondrex, WA, USA) emulsified in Freund’s complete adjuvant (Sigma). A booster immunization of chicken type II collagen emulsified in Freund’s incomplete adjuvant (Sigma) was used 21 days after the primary immunization. The mice were randomly divided into 4 groups: A. normal group: mice treated with saline (n = 10); B. control group: CIA mice treated with saline (n = 15); C. 10 mg/kg group: CIA mice treated with MASM 10 mg/kg (n = 15); and D. 30 mg/kg group: CIA mice treated with MASM 30 mg/kg (n = 15). The *in vivo* dosage of MASM were selected based on previous publications investigating the pharmaceutical properties of MASM^17, 19^. Thereafter, the mice were closely monitored and scored every 2 days in a blinded manner for signs of arthritis. The arthritis severity was scored on a scale from 0 to 4 by visual evaluation of each paw as follows: 0, normal paw; 1, redness and mild swelling confined to the tarsals or ankle joint; 2, redness and mild swelling extending from the ankle to the tarsals; 3, redness and moderate swelling extending from the ankle to metatarsal joints; and 4, redness and severe swelling encompass the ankle, foot and digits, or ankylosis of the limb^46^. Hind paw thickness was measured with an electric caliper placed across the ankle joint at the widest point. The paw thickness index was defined as the increase in the diameter of the arthritic ankle at specific time points over the diameter determined on day 21, and this value is presented as a percentage. Starting on day 22, mice were orally treated with saline or different doses of MASM. On day 42, the mice were killed, and then the serum and joint tissues were harvested for further assessment.

### Micro–computed tomography (micro-CT) imaging

A SkyScan 1176 micro-CT apparatus was used to evaluate structural changes in the hind paws. The hind paws (from the tip of the toes to the mid of the tibia) obtained from experimental mice were scanned and reconstructed into a 3-dimensional structure with a voxel size of 9 μm. The projection images were reconstructed into 3-dimensional images using the NRecon software (version 1.6.1.5) and a CT Analyser (Version: 1.15.4.0 + , Bruker, Kontich, Belgium).

### Histopathological assessment

The mice were killed and joint tissues were skinned and fixed in 4% buffered formaldehyde, then decalcified in 12% EDTA for 15 days. The tissues were then dehydrated, paraffin embedded, sectioned, and stained with hematoxylin and eosin. The extent of synovitis, pannus formation, and destruction of bone and cartilage was determined using a graded scale: grade 0, no signs of inflammation; grade 1, mild inflammation with hyperplasia of the synovial lining and minor cartilage damage; grades 2 through 4, increasing degrees of inflammatory cell infiltrate and destruction of bone and cartilage. Histopathological analyses were performed using microscopy (Olympus, Tokyo, Japan).

### Immunohistochemical staining of joint tissues

For local TNF-α staining in joint tissues, deparaffinized sections were subjected to antigen retrieval in 0.01 M citrate buffer solution, pH 6.0, at 100 °C for 15 minutes. Rabbit anti-mouse TNF-α polyclonal antibody (Abcam, Cambridge, UK) was used as a primary antibody, and horseradish peroxidase–conjugated goat anti-rabbit antibody was used as a secondary antibody.

### Cytokine assays

Mice serum samples were prepared after they were incubated under ice-cold conditions and then centrifuged for 10 min (1000× *g*). The samples were stored at −80 °C until analysis. Then, the serum levels of TNF-α, IL-1β, IL-6 were determined using commercial ELISA kits (Arigo Biolabratories, Taiwan) according to the manufacturer’s protocols and instructions.

### Isolation and culture of human RA-FLS

RA-FLS were isolated from human synovial tissues, which were obtained from patients with RA who met the diagnostic criteria of the American College of Rheumatology^47^ and had undergone total knee replacement. Synovial tissues were minced and digested with 10 μg/ mL collagenase in serum-free Dulbecco’s modified Eagle’s medium (DMEM, Gibco Invitrogen) for 2 h at 37 °C. Then, the cell suspensions were filtered through sterile 70-μm nylon cell strainers (BD Biosciences, San Jose, CA, USA), washed, and cultured in DMEM supplemented with 10% fetal bovine serum (FBS), 1% penicillin/streptomycin, and 1% L-glutamine (all reagents from Invitrogen). The cells were grown at 37 °C under a humidified atmosphere containing 5% CO_2_. The RA-FLS from passages 3–8 were used for all experiments. All treatments were performed in a serum-free medium. Informed consent was obtained from all patients, and the study protocol was approved by the Ethics Committee of Changhai hospital, Second Military Medical University.

### Cell viability assay

Cell viability was determined using the Cell Counting Kit-8 (CCK8) (Dojindo, Kumamoto, Japan) according to the manufacturer’s instructions. In brief, RA-FLS cells in logarithmic growth-phase were collected, 6 × 10^3^/well cells were seeded in a 96-well plate with 100 μL culture medium. After 24 h of culture, various concentrations of MASM (0, 1, 5, 10, 20, 40, 80 μM) were put in different wells. Each of the concentrations above was regarded as one treatment group, while there was no MASM in the control group. Each treated or control group contained five parallel wells. The culture plates were then incubated for 12 h, 24 h, and 48 h, and 10 µl of the tetrazolium substrate were added to each well of the plate. The Plates were incubated at 37 °C for 1 h, then the optical density (OD) was measured at 450 nm using a microplate reader. The cell viability was calculated according to the following equation: the cell viability = (OD experiment − OD blank)/(OD control − OD blank) × 100%.

### Quantitative RT-PCR analysis

Total RNA was extracted using TRIzol reagent (Invitrogen, Carlsbad, CA, USA) according to the manufacturer’s instructions, and 1 μg was reverse transcribed using PrimeScript reagent Kit (Takara, Dalian, China). PCR amplification was performed in triplicate on a StepOnePlusTM real-time PCR system (Applied Biosystems, USA) using the SYBR Premix Ex TaqTM PCR Kit (Takara, Dalian, China). β-actin was amplified as an internal control. The levels of assayed mRNAs were calculated with comparative Ct method and were normalized to an un-treated control.

### Induction and detection of apoptosis

RA-FLS were pretreated with different concentrations of MASM for an hour then co-incubated with 2 ng/ml IL-1β for 48 hours. Apoptosis was detected using the Annexin V-FITC/PI Apoptosis Detection kit (BD biosciences, San Jose, CA, USA) and analyzed by flow cytometry with a FACSCalibur flow cytometer (BD Biosciences, San Jose, CA).

### Analysis of Mitochondrial Membrane Potential (Δψm)

Δψm was analyzed by the fluorescent probe JC-1 using flow cytometry. Pretreated RA-FLS were stained with JC-1 (5,5′,6,6′-tetrachloro-1,1′,3,3′ tetraethyl benzimidazole carbocyanine iodide; JC-1 Mitochondrial Membrane Potential Assay Kit, Cayman Chemical) for 15 min at 37 °C, 5% CO_2_, and then washed with phosphate-buffered saline (PBS) and analyzed using flow cytometry with a FACSCalibur flow cytometer (BD Biosciences, San Jose, CA) according to the manufacturer’s instructions.

### Preparation of cytoplasmic and nuclear protein extracts

RA-FLS were pretreated with different concentrations of MASM in 100-mm dishes for an hour and then co-stimulated with 2 ng/ml IL-1β for 30 mins. Cells were harvest with trypsin-EDTA and then centrifuged at 500× g for 5 minutes before the cells were washed twice by centrifugation in PBS 500× g for 5 minutes. Cytoplasmic and nuclear extracts were prepared from the pellets using NE-PER nuclear and cytoplasmic protein extraction reagents (Thermo Scientific) according to the protocol provided by the manufacturer.

### Western blot analysis

RA-FLS were homogenized in RIPA buffer with protease and phosphatase inhibitors (Calbiochem). The homogenates, which contained 25 μg of protein, were separated by 12% sodium dodecyl sulfate–polyacrylamide gel electrophoresis and transferred to 0.22 μm polyvinylidene difluoride (PVDF) membranes. The membranes were blocked with 5% non-fat milk in TBS/Tween (0.5 M NaCl and 20 mM Tris pH 7.5 with 0.1% (v/v) Tween-20) at room temperature (RT) for 2 hours. The membranes were incubated with primary antibodies diluted in 5% BSA in TBS/Tween buffer overnight at 4 °C, washed with TBS/Tween and incubated with the appropriate horseradish peroxidase (HRP)-conjugated secondary antibody in 5% non-fat milk in TBS/Tween for 1 hour at RT. The membranes were then washed with TBS/Tween and the protein bands were visualized using the ECL detection system. Quantification of the bands was performed using the ImageJ software (National Institute of Health, NIH).

### Statistical analysis

Data are reported as the mean and standard error of the mean (SEM). Statistical comparisons were performed using one-way ANOVA, followed by Dunnett’s multiple comparisons analysis (between different groups). P < 0.05 were considered to be significant.

## Electronic supplementary material

Supplementary file
