# Supplementary material for: Therapeutic effects of matrine derivate MASM in mice with collagen-induced arthritis and on fibroblast-like synoviocytes
Source: Sci Rep. 2017 May 26;7:2454. doi: 10.1038/s41598-017-02423-7 (PMC5446426; doi:10.1038/s41598-017-02423-7)

Therapeutic effects of matrine derivate MASM in mice with collagen-induced arthritis and on fibroblast-like synoviocytes

YumingZou[1†], MD, Quan Li[1†], MD, PhD, Denghui Liu[1], MD, PhD, Jia Li[1], MD, PhD, Qing Cai[3], MD, PhD, Chao Li[2], Qingjie Zhao[2*], PhD, WeidongXu[1*], MD, PhD

1. Department of Orthopedics, Changhai hospital, the first affiliated hospital of the Second Military Medical University, Shanghai 200433, P.R. China
2. Department of Organic Chemistry, School of Pharmacy, Second Military Medical University, Shanghai 200433. P.R. China. Email: qjzhao@smmu.edu.cn
3. Department of Rheumatology, Changhai hospital, the first affiliated hospital of the Second Military Medical University, Shanghai 200433, P.R. China. Email: xwdsmmu@163.com

[†] These authors contributed equally to this work.

* Corresponding to Weidong Xu or Qingjie Zhao.

Figure S1. Larger number of regional enlarged representative photographs of 3D reconstruction of micro-computed tomography of the hind paws (right) of mice with CIA.

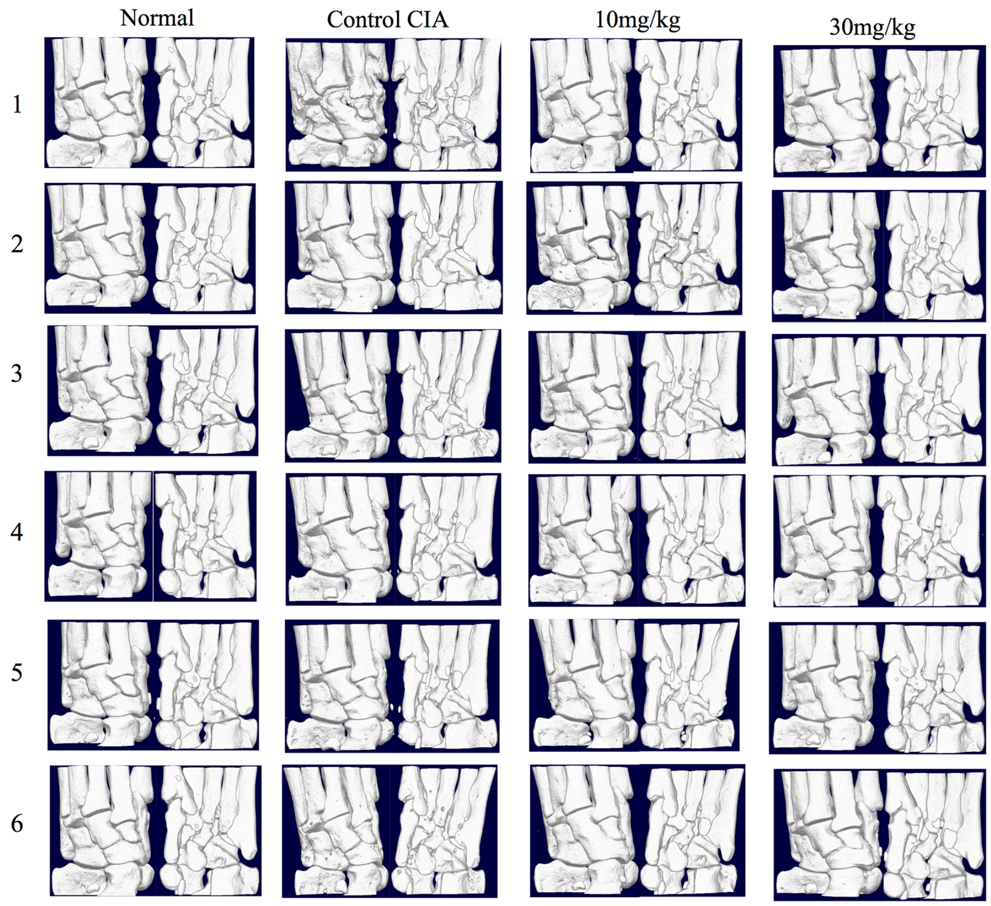

Figure S2. Representative photographs of hematoxylin and eosin (H&E) staining of the hind paws.

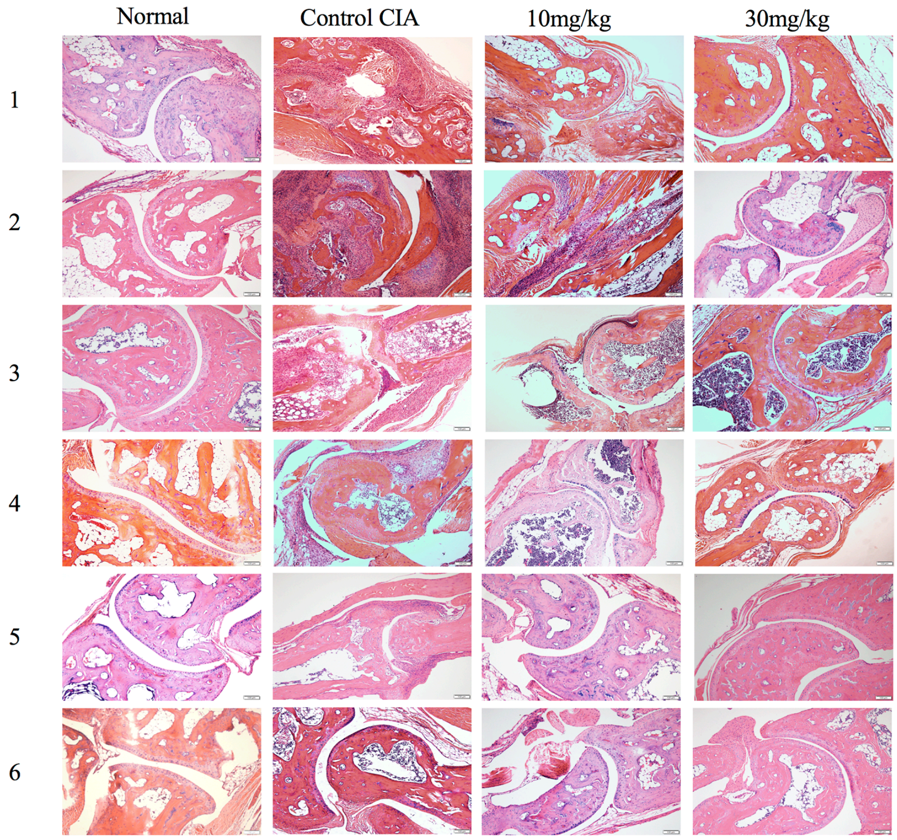

Supplement: Supplementary file 1 — Supplementary file [file 41598_2017_2423_MOESM1_ESM.pdf]
